# Combined analysis of cecal microbiota and metabolomics reveals the intervention mechanism of Dayuan Yin in acute lung injury

**DOI:** 10.3389/fphar.2024.1436017

**Published:** 2024-09-10

**Authors:** Lei Zhang, Wei Zhu, Zepeng Zhang

**Affiliations:** Kunshan Hospital of Traditional Chinese Medicine, Kunshan, China

**Keywords:** acute lung injury, Dayuan Yin, metabonomics, intestinal flora, inflammatory factors, Pearson analysis

## Abstract

The ancient Chinese medicinal formula, Dayuan Yin (DYY), has a long history of use in treating respiratory ailments and is shown to be effective in treating acute infectious diseases. This study aims to explore how DYY may impact intestinal flora and metabolites induced by acute lung injury (ALI). ALI rats were induced with lipopolysaccharide (LPS) to serve as models for assessing the anti-ALI efficacy of DYY through multiple lung injury indices. Changes in intestinal microflora were assessed via 16SrRNA gene sequencing, while cecum contents were analyzed using non-targeted metabonomics. Differential metabolites were identified through data analysis, and correlations between metabolites, microbiota, and inflammatory markers were examined using Pearson’s correlation analysis. DYY demonstrated a significant improvement in LPS-induced lung injury and altered the composition of intestinal microorganisms, and especially reduced the potential harmful bacteria and enriched the beneficial bacteria. At the gate level, DYY exhibited a significant impact on the abundance of Bacteroidota and Firmicutes in ALI rats, as well as on the regulation of genera such as Ruminococcus, *Lactobacillus*, and Romboutsia. Additionally, cecal metabonomics analysis revealed that DYY effectively modulated the abnormal expression of 12 key metabolic biomarkers in ALI rats, thereby promoting intestinal homeostasis through pathways such as purine metabolism. Furthermore, Pearson’s analysis indicated a strong correlation between the dysregulation of intestinal microbiota, differential metabolites, and inflammation. These findings preliminarily confirm that ALI is closely related to cecal microbial and metabolic disorders, and DYY can play a protective role by regulating this imbalance, which provides a new understanding of the multi-system linkage mechanism of DYY improving ALI.

## 1 Introduction

Acute lung injury (ALI) is a severe inflammatory response that damages lung cells and reduces their function (Lu et al., 2022), with a mortality rate of 30%–40% (Zheng et al., 2022; Li et al., 2023). Research shows that inflammatory dysregulation is key in ALI, but gaps remain in understanding of its causes and regulation (Millar et al., 2022). TCM theory links ALI to dyspnea syndrome and chest knot, caused by external and internal factors like diet, emotion, fatigue, or illness. The theory of the “lung and large intestine are interiorly exteriorly related” suggests that pathological changes in extrapulmonary viscera may contribute to the occurrence of ALI (Ding et al., 2020). Current treatments such as corticosteroids have not been effective in the long term (Dushianthan et al., 2011), so new drugs are urgently needed to improve and treat ALI. Supportive interventions like oxygen and mechanical ventilation may have limited efficacy and could potentially worsen lung function or cause permanent injury (Mokrá, 2020; Bezerra et al., 2023; Lin et al., 2020).

Traditional Chinese medicine (TCM) shows promise in preventing and treating ALI by targeting different biological pathways (Gao et al., 2018; Li et al., 2024), but there are still complex issues that cannot be explained using traditional methods. Metabolomics can be used to analyze metabolic profiles during disease and treatment, aligning with TCM overall thinking (Wang et al., 2017; Saoi and Britz-McKibbin, 2021), aiding in improving traditional practices and drug development (Wang et al., 2015) and enhancing the understanding of the pharmacological effects and therapeutic mechanisms of active metabolites in traditional Chinese medicine, aiding in the discovery and development of new drugs for complex diseases (Wang et al., 2021).

In addition, the human intestine has a complex microbiota and plays a crucial role in processes like energy metabolism, immune regulation, and digestion (Khan et al., 2014); the gut microbiota (GM) forms a sensitive and delicate symbiotic relationship with the human body. Therefrom, imbalance in the GM can negatively impact health and contribute to various diseases (Guo et al., 2019; Moreno-Indias et al., 2021), including the development of respiratory diseases (Li et al., 2019). Intestinal and lung activities are connected through flora, metabolites, and mucosal immunity, which is how the gut–lung axis demonstrates works (Dang and Marsland, 2019; He et al., 2022). Botanical drugs may help restore intestinal balance and improve gut health, offering a new approach in treating lung diseases (He et al., 2022; Lu et al., 2024).

DYY is a traditional prescription with a long history of use, first proposed by Wu Youke in the Ming Dynasty (Ruan et al., 2020). It contains seven botanical drugs, namely, *Magnolia officinalis*, *Amomum tsao-ko*, Areca nut, *Scutellaria baicalensis*, *Anemarrhena asphodeloides* Bunge, Radix paeoniae Alba, and Radix Glycyrrhizae, and is used to treat epidemic diseases like SARS (Zhang, 2003; Huang and Yan, 2005). DYY’s prescription drugs contain metabolites with various beneficial effects for treating lung diseases (Sharawi et al., 2024), such as biphenyl neolignans (Wang et al., 2023), saponins (Park et al., 2018), and flavonoids (Tsai et al., 2014; Zhu et al., 2023). Previous studies suggest its polyphenol metabolites can help with ALI (Guo et al., 2023), but more research is needed to understand how it works. This study analyzed how DYY plays its anti-ALI effect and affects cecal flora and metabolites.

## 2 Materials and methods

### 2.1 Drugs and reagents

The botanical drugs of DYY are betel nut (*Areca catechu* L., BL), *Magnolia officinalis* (*Magnolia officinalis* Rehd.et Wils., HP), *Amomum tsao-ko* (*Amomum tsao-ko* Crevost et Lemaire, CG), *Anemarrhena asphodeloides* (*Anemarrhena asphodeloides* Bge., ZM), Radix paeoniae Alba (Paeonia lactiflora Pall., BS), *Scutellaria baicalensis* (*Scutellaria baicalensis* Georgi, HQ), and licorice (*Glycyrrhiza glabra* L., GC), and the batch numbers of Chinese medicines are 220924, 220905, 220330, 220912, 220930, and 221010. All the medicinal materials were purchased from Tianling Traditional Chinese Medicine Co., Ltd. (Suzhou, China) and identified by pharmacy experts. Lipopolysaccharide (LPS) is supported by Beijing Solebo (batch number: MB5198). Dexamethasone sodium phosphate injection (DXM) was purchased from Chenxin Pharmaceutical Co., Ltd. (Shandong, China, batch number: 23011011). Analytical-grade methanol (batch number: CAEQ-4-003302-4000), acetonitrile (batch number: CAEQ-4-003306-4000), and formic acid (batch number: 4.014784.0500) are provided by ANPEL (Shanghai, China). Ultra-pure water was prepared by using Milli-Q plus system (Milibo, Massachusetts, United States).

### 2.2 Preparation of DYY

BL 7.46 g, HP 3.73 g, CG 1.87 g, ZM 3.73 g, BS 3.73 g, and GC 1.87 g; a total of 26.12 g was weighed according to the composition ratio of DYY, 10 times distilled water was added, boiled at 100°C for 60 min, filtered, decocted in the same way, the decoctions were combined, and the DYY group was concentrated to 50 mL by using a rotary evaporator.

### 2.3 Animal treatment and sample collection

In this study, 24 SPF-grade male Sprague–Dawley rats, weighing (200 ± 20) g, were selected and provided by Pengyue Experimental Animal Breeding Co., LTD. (Jinan, China). The experimental procedure was approved by the Experimental Animal Welfare Ethics Committee of Institute of Biology, Shandong Academy of Sciences (SWS20240130). Before ALI modeling, all rats were kept under the conditions of relative humidity (50 ± 5) %, temperature (24 ± 2) °C, alternating light and dark cycles each for 12 h, and were provided with *ad libitum* access to food and water for 1 week. Subsequently, all rats were randomly divided into four groups (six rats per group), namely, the control group (Con), model group (LPS), model + dexamethasone group (LPS + DXM), and model + DYY group (LPS + DYY). First, except the Con group, the other groups of rats were intraperitoneally injected 10 mg·kg^−1^ LPS to establish the ALI model. After 6 h of modeling, the modeling was confirmed by symptoms such as shortness of breath, tracheal rales, restlessness, and rapid heart rate in rats. Based on the dose conversion between rats and humans, the LPS + DYY group was given twice the clinical equivalent dose of DYY (4.70 g·kg^−1^) once a day for 1 week, LPS and Con groups were given aseptic normal saline intragastrically, and the LPS + DXM group was injected intraperitoneally with the maximum clinical equivalent dose of DXM (2 mg·kg^−1^) at days 1, 4, and 7, respectively. Twelve hours after the last treatment, all rats were killed and bronchoalveolar lavage fluid (BALF) and lung tissue were collected. The cecum of rats was washed with cooled Milli-Q water to collect the contents and stored at −80°C for further examination.

### 2.4 Evaluation of lung injury in rats

#### 2.4.1 Determination of inflammatory factors in BALF

Cervical surgery was performed on the rats to expose the trachea. The left lung was washed with 0.5 mL PBS solution three times, and the BALF was centrifugated for collecting the supernatant. Interleukin-6 (IL-6) (batch number: ml064292), interleukin-6 (IL-1β) (batch number: ml037361), and tumor necrosis factor- α (TNF-α) (batch number: ml002859) ELISA kits were purchased from Enzyme-linked Biotechnology Co., Ltd. (Shanghai, China) and used to measure inflammatory cytokine levels.

#### 2.4.2 Determination of lung wet-to-dry (W/D) weight ratio

The lower leaf of the right lung of rats was taken, cleaned with ultra-pure water, the surface moisture of the lung was absorbed by using a filter paper, the lung was weighed and labeled as wet weight, and then the lung was placed in a constant temperature drying oven at 60°C for 48 h. The lung was weighed again and labeled as dry weight, and the W/D weight ratio of the lung tissue was calculated.

#### 2.4.3 Evaluation of the pathological morphology of the lung tissue

The upper lobe of the right lung of rats in each group was fixed with 4% paraformaldehyde solution for 24 h, embedded in paraffin wax and cut into 4-μm-thick sections, stained with hematoxylin-eosin (H&E), sealed, and placed under an optical microscope for photography and observation. The degree of lung injury was blindly evaluated by Smith score (Smith et al., 1997), including pulmonary edema, alveolar, and interstitial inflammatory cell infiltration, hemorrhage, and atelectasis, among which 0 points indicated no damage; 1 points indicated the lesion range <25%; 2 points indicated the lesion range is 25%–50%; 3 points indicated the lesion range is 50%–75%; 4 points indicated the lesion range >75%.

### 2.5 Study on intestinal flora

#### 2.5.1 16S rDNA sequencing

DNA was extracted from the cecal contents of different samples (n = 4) using the E.Z.N.A.®StoolDNAKit (Omega, Inc., America, D4015-00) according to the manufacturer’s instructions. The quality of the resulting DNA was measured using 1% agarose gel electrophoresis, eluted with 50 μL elution buffer, and stored at −80°C. Primers 341F(CCTAYGGGRBGCASCAG) and 806R (GGACTACNNGGGTATCTAAT) were used to amplify the V3–V4 variable region of the 16SrRNA gene by PCR. The final product was confirmed by 2% agar-gel electrophoresis, and the electrophoretic map was detected by a 3-μL sample. PCR products were purified and quantified by AxyPrep DNA Gel Extraction (Corning, Inc., America, AP-GX-50) and Quantus™ Fluorometer (Promega Biotech Co., Ltd., Beijing, China), respectively. According to the sequencing of each sample size requirements, it will be mixed according to a certain proportion of different samples of PCR products, and then the TruSeq Nano DNA LT library Prep Kit build Illumina was used for library preparation. AmpliCon library is sequenced and read by the Illumina NovaSeq PE250 platform (Lv et al., 2021).

#### 2.5.2 Data processing and analysis

We used Trimmomatic, Pear, and Flash to filter raw reads, that ensured high-quality data. VSEARCH screened mosaic sequences, while DADA2 obtained feature lists and sequences. Operational taxonomic units (OTUs) were identified with 97% similarity to the Greengenes database. Venn diagrams illustrated species differences between groups. Data were normalized and analyzed with QIIME2, using Alpha diversity metrics to assess microorganism abundance and diversity. Beta diversity analyses, including principal coordinate analysis (PCoA) and non-metric multidimensional scaling analysis (NMDS), were used to visualize community structure changes. Linear discriminant analysis of effect size (LEfSe) identified potential markers of differences among microbiota groups, with the top 10 phyla and 30 genera selected for classification analysis (Gao et al., 2023). Microbiota function was predicted using PICRUSt software.

### 2.6 Metabolomics studies

#### 2.6.1 Sample preparation and extraction

After the cecal samples were naturally thawed, 50 mg of cecal contents was mixed with pre-cooled water and methanol–acetonitrile solution (1:1, v/v), vortexed for 60 s, and subjected to ultrasonic extraction for 30 min. The protein was precipitated at −20°C for 1 h, centrifuged at 4°C at 12000 rpm for 10 min, the supernatant was vacuum-dried, and dissolved in acetonitrile before being transferred to a vial.

#### 2.6.2 Cecal metabolomics analysis

The chromatography was performed on Waters UPLC HSST3 column (2.1 mm × 100 mm, 1.8 μm) with mobile phase: 0.1% formic acid water (A) −0.1% formic acid acetonitrile (B), gradient elution (0 ∼ 1 min, 0% B; 1–9 min, 0% B→ 95%B; 9–13 min, 95%B; 13–13.1 min, 95%B→0%B; 13.1–17 min, 0%B), the flow rate was 0.3 mL/min, the column temperature was 40°C, and the sample size was 2 μL. Metabolites eluted from chromatographic columns were detected on a Q Exactive HFX mass spectrometer equipped with a heated ESI source, operating in positive and negative ion modes. ESI source parameters are set as follows: spray voltage, −2.8 kV/3.0 kV; nebulizer temperature, 350°C; sheath gas pressure, 40 Arb; auxiliary pressure, 10 Arb; ion transfer tube temperature, 320°C, full-MS-ddMS2 scanning mode, and scanning range of 70–1050 m/z. In order to evaluate the stability and repeatability of LC-MS during the entire collection process, one QC sample was collected regularly after every 10 samples for analysis (Chen et al., 2020).

#### 2.6.3 Data processing and multivariate analysis

Progenesis QI software (Waters Corporation, Milford, United States) was used to preprocess the data, resulting in a data matrix file with metabolite information, such as metabolite retention time (t_R_), mass-charge ratio, and peak intensity. The data included variables with a non-zero value of more than 80% in any sample and a QC sample relative standard deviation (RSD) < 30% after normalization. The data were then analyzed using SIMCA14.0 (Umetrics, Sweden). Principal component analysis (PCA) was used for sample clustering and trend analysis, partial least squares discrimination analysis (PLS-DA) for data dimension reduction and visualization of group differences, and orthogonal partial least squares discriminant analysis (OPLS-DA) to minimize differences among groups (Li et al., 2022). Volcanic maps were used to identify significant differential metabolites based on criteria including variable importance (VIP) value > 1 and *p* < 0.05 (from the two-tailed *t* test) and fold change (FC) value >1.5 or FC < 0.67. Create metabolite heat maps for clustering analysis, utilize HMDB gene and genome (http://www.hmdb.ca/) and Kyoto Encyclopedia of Genes and Genomes (KEGG) database (http://www.kegg.ca/) to identify and annotate fragment ions, and the retrieval error was set to 0.1 (Yu et al., 2021). Then, we screened for metabolic pathways with influence value >0.05 using MetaboAnalyst 5.0 for metabonomic pathway analysis (MetPA) to identify potential target pathways.

### 2.7 Statistical analysis

SPSS software (version 26.0, United States) was used to analyze the data by one-way ANOVA, and the results are expressed as mean ± SD. Student’s unpaired t test was used to identify significant treatment differences. Pearson’s correlation analysis was performed to determine the relationship between variables (Lv et al., 2021). *p* < 0.05 is considered to be statistically significant.

## 3 Results

### 3.1 DYY alleviates lung injury in LPS-induced ALI rats

**FIGURE 1:**
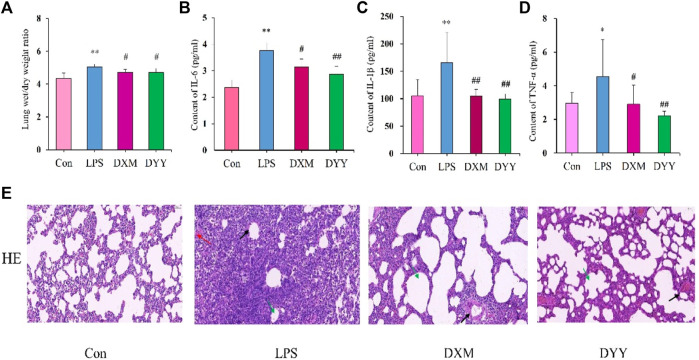
Trends in change in the Con, LPS, DXM, and DYY groups during the establishment of the ALI model (n = 6). ∗*p* < 0.05, ∗∗*p* < 0.01 compared to the Con group; #*p* < 0.05, ##*p* < 0.01 compared to the LPS group. **(A–D)** Changes in W/D ratio, IL-6, IL-1β, and TNF-α; **(E)** H&E staining results of lung tissue (200×). The black, red, and green arrows in the LPS group indicated an increase in the thickening of the alveolar septum, an infiltration of inflammatory cells, and a smaller alveolar cavity, respectively.

ALI is marked by increased alveolar/capillary permeability, inflammation, and tissue damage (Yu et al., 2021). In this study, histopathology confirmed successful ALI modeling by LPS, showing infiltration of inflammatory cells, thickening of the alveolar septum, and smaller alveolar cavity (Figure 1E). Rats in the LPS group showed significantly higher lung W/D ratio and levels of inflammatory markers (IL-6, IL-1β, and TNF-α) in BALF compared to the Con group (*p* < 0.01 or *p* < 0.05), indicating excessive inflammatory response in lung tissue, but that in DXM and DYY groups were significantly decreased (*p* < 0.01 or *p* < 0.05) (Figures 1A–D), indicating that DYY has a therapeutic effect on ALI by reducing inflammation and pulmonary edema. The results also showed that the Smith score of the LPS group was significantly higher than that of the Con group, and the Smith score of the DYY group was significantly lower than that of the LPS group. See Table 1.

**TABLE 1:** Comparison of Smith score of lung injury in rats of each group (x ± s, n = 6).

Group	Smith score	95% confidence interval	
		Lower limit	Upper limit
Con	0.45 ± 0.14	0.3	0.7
LPS	3.65 ± 0.19^a^	3.4	3.9
DYY	2.50 ± 0.34^b^	2.1	3.0
DXM	1.70 ± 0.18^b^	1.5	2.0

Note: compared with the Con group.

^a^ *p* < 0.01; compared with LPS, group.

^b^ *p* < 0.01.

### 3.2 DYY regulates intestinal ecological imbalance in LPS-induced ALI rats

**FIGURE 2:**
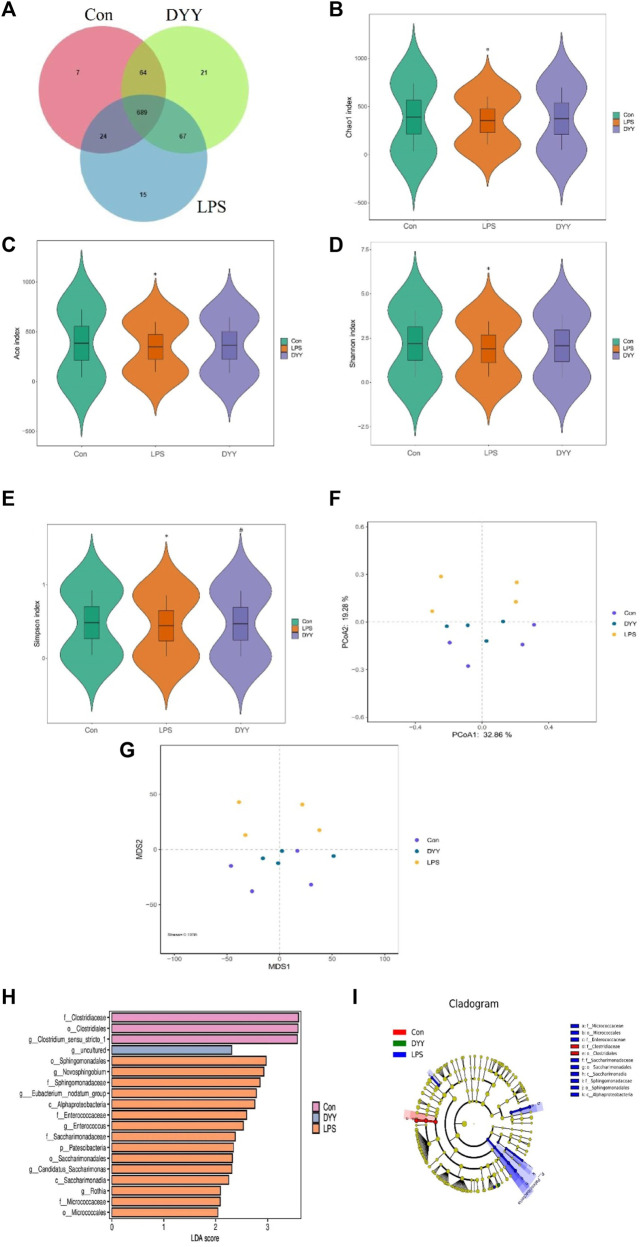
**(A)** Venn diagram depicting the distribution of OTUs among different groups. **(B–E)** Alpha diversity of intestinal flora and analysis of beta diversity **(F, G)**. **(H)** LDA effect size analysis of the major biomarker taxa. **(I)** Cladogram obtained from LEfSe analysis. ∗*p* < 0.05 compared to the LPS group and #*p* < 0.05 compared to the Con group.

Venn diagram (Figure 2A) shows DYY partially restored LPS-induced intestinal flora disorder in rats, with 753 OTUs shared by DYY and Con and 713 OTUs shared by LPS and Con.). DYY intervention increased α-diversity and restored balance in intestinal flora, particularly the Simpson index (*p* < 0.05) (Figures 2B–E). Beta diversity was also studied to analyze overall microbial community structure. PCoA and NMDS analyses showed significant separation between Con and LPS samples, with DYY samples distributed similarly to Con samples (Figures 2F,G), indicating that LPS disrupted the cecal microbial community but DYY helped restore balance in rats.

We used LEfSe to identify biomarkers with LDA >2 and *p* < 0.05 to assess the impact of DYY intervention on rat gut bacteria. Nineteen different species were significantly abundant among the three groups (Figure 2H). Clostridiaceae, Clostridiales, and *Clostridium*_sensu_stricto_1 were more abundant in the Con group, while Sphingomonadales, Novosphingobium, Sphingomonadaceae, and Eubacterium_nodatum_group were more abundant in the LPS group. DYY treatment increased the abundance of uncultured bacteria, indicating they may be intestinal markers of DYY, improving LPS-induced ALI. The branching diagram further illustrates the specific intestinal microflora associated with DYY therapy (Figure 2I).

**FIGURE 3:**
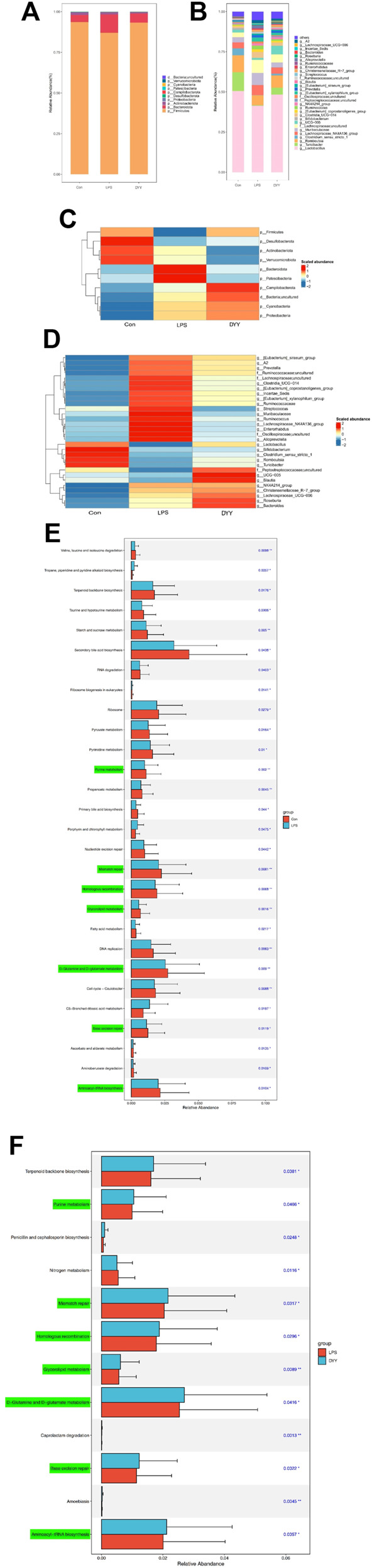
**(A)** Species composition abundance map at the phylum and the genus levels **(B)**. **(C)** Abundance clustering heat map of the phylum and the genera **(D)**. In the heat map, the closer to blue, the lower the abundance, and the closer to red, the higher the abundance. The length of the bar of the LDA represents the influence of species abundance on the different effects. **(E)** KEGG pathways in Con and LPS groups. **(F)** LPS and DYY groups were enriched. The green mark on the left side was marked as the pathway significantly changed by both groups.

Differences in dominant gates were found between the Con and LPS groups, with Bacteroidota and Patescibacteria increasing and Firmicutes and Desulfobacterota decreasing in the LPS group (Figure 3A). At the genus level, uncultured Lachnospiraceae, [Eubacterium]_xylanophilum_group, Ruminococcus, NK4A214_group, and Enterorhabdus were more abundant in the LPS group, while *Lactobacillus*, Turicibacter, Romboutsia, and UCG-005 were less abundant (Figure 3B). DYY treatment reversed these abundance levels of bacterial phyla and genera affected by LPS, as shown in the heat map (Figures 3C,D). This further suggests that DYY partially improved the ecological imbalance of the phylum and genus in rats induced by LPS.

We also used PICRUSt analysis to predict gut microbiota function based on the KEGG database. The LPS group showed increased expression of metabolic pathways like tropane, piperidine, pyridine alkaloid biosynthesis, porphyrin, and chlorophyll metabolism compared to the Con group. However, certain metabolic pathways like purine metabolism, mismatch repair, homologous recombination, D-glutamine and D-glutamate metabolism, base excision repair, and glycerolipid metabolism were lower in the LPS group compared to the Con group, and these pathways showed their abundance was reversed after DYY intervention (Figures 3E,F).

### 3.3 DYY regulates the abnormal level of cecal metabolites in LPS-induced ALI rats

**FIGURE 4:**
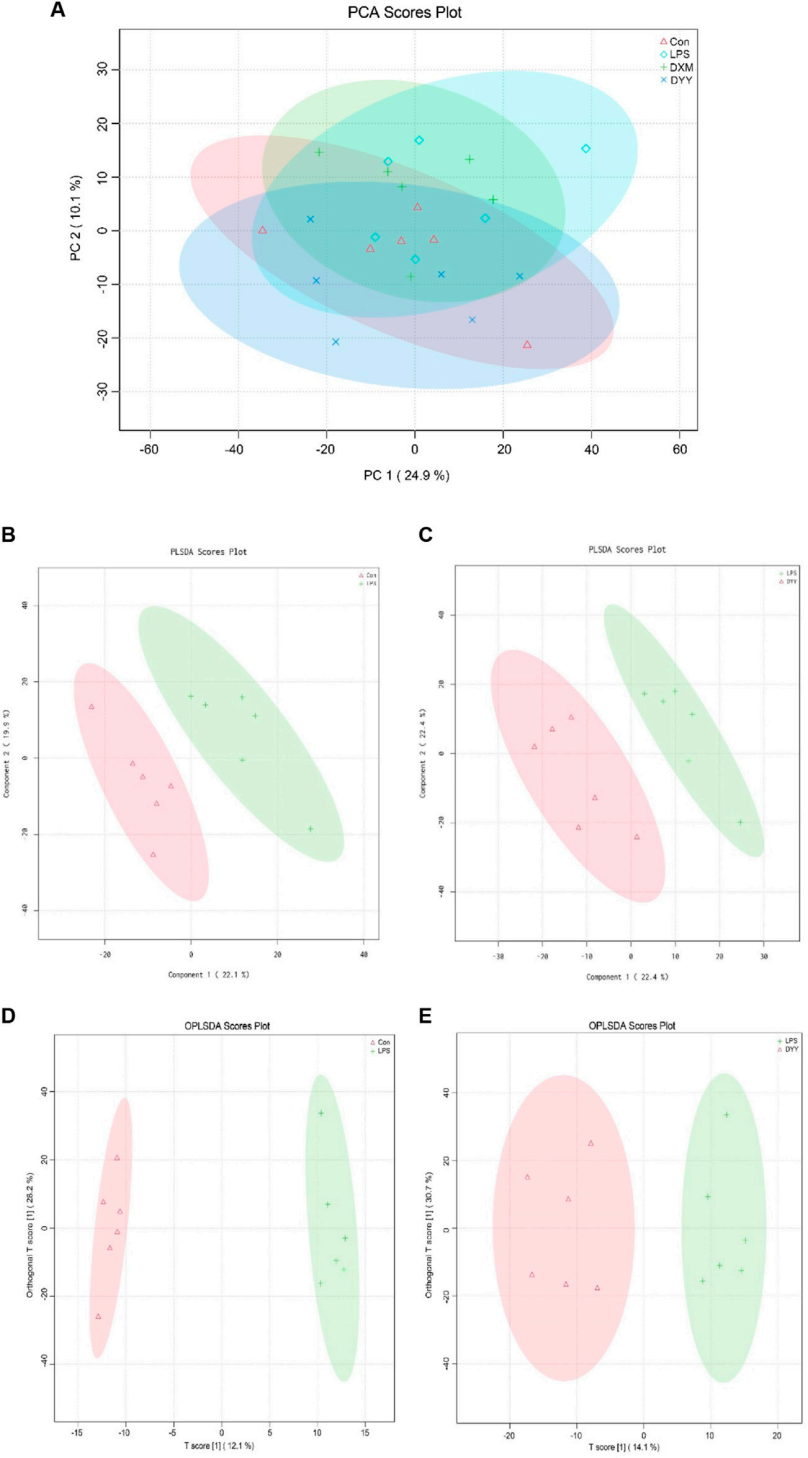
**(A)** PCA score scatter diagram, **(B–C)** PLS-DA score scatter diagram, and **(D, E)** OPLS-DA score scatter diagram of Con , LPS , and DYY groups in the total ion mode.

The total ion chromatography (TIC) of quality control (QC) samples showed good peak shape and uniform distribution with a stable detection system (Supplementary Figure S1). PCA revealed a distinct separation between Con and LPS groups, indicating significant changes in endogenous metabolites of ALI rats. DYY group samples were closer to the Con group (Figure 4A). PLS-DA results showed metabolite differences or similarity among LPS, Con, and DYY groups, suggesting DYY may help regulate abnormal metabolism in ALI rats (Figures 4B,C).

**FIGURE 5:**
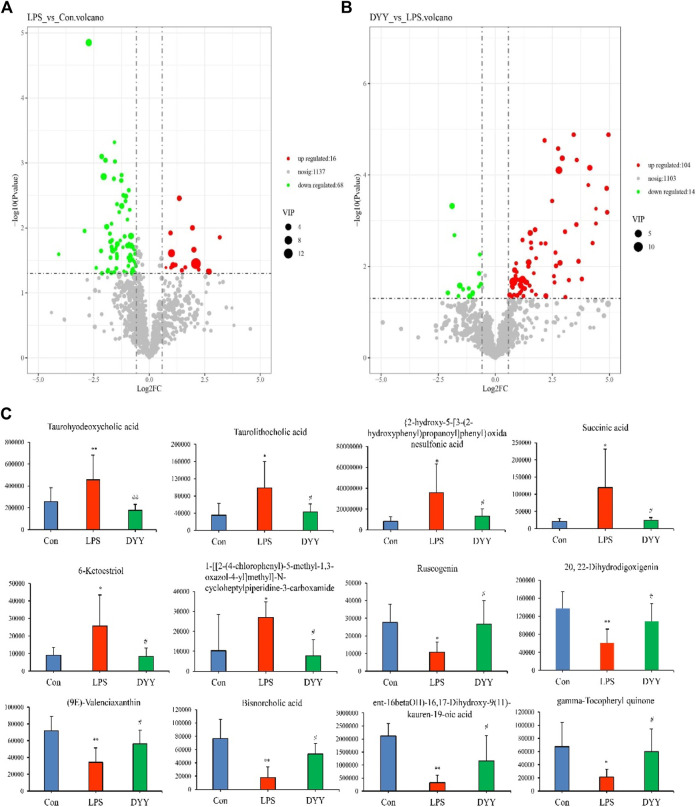
**(A)** Volcano plot map in the total ion mode of Con and LPS, **(B)** LPS, and DYY. **(C)** Twelve metabolites with significantly differential abundance.

OPLS-DA was used to distinguish between the Con group, LPS group, and DYY group, showing significant aggregation within each group and a clear separation between groups (Figures 4D,E). Important variables were identified in volcano maps (Figures 5A,B), and differential metabolites were identified through cluster analysis of heat maps (Supplementary Figure S2). The results showed that compared with the Con group, six metabolites in the LPS group were significantly upregulated, namely, taurohyodeoxycholic acid, taurolithocholic acid, {2-hydroxy-5-[3-(2-hydroxyphenyl)propanoyl]phenyl}oxidanesulfonic acid, succinic acid, 6-ketoestriol, and 1-[[2-(4-chlorophenyl)-5-methyl-1,3-oxazol-4-yl]methyl]-N-cycloheptylpiperidine-3-carboxamide. Six metabolites were significantly downregulated, including ruscogenin, 20,22-dihydrodigoxigenin, (9e)-valenciaxanthin, bisnorcholic acident-16beta (OH)-16,17-dihydroxy-9 (11)-kauren-19-oic acid, and gamma-tocopheryl quinone. In the DYY group, these potential biomarker levels returned to normal (Figure 5C), indicating DYY restores LPS-induced abnormal levels of metabolites *in vivo*, affecting the biosynthesis and purine metabolism of secondary metabolites that may be associated with ALI, such as the biosynthesis of isoflavones (Supplementary Figure S3).

### 3.4 Correlation among cecal microbiota, differential metabolites, and inflammatory indicators

**FIGURE 6:**
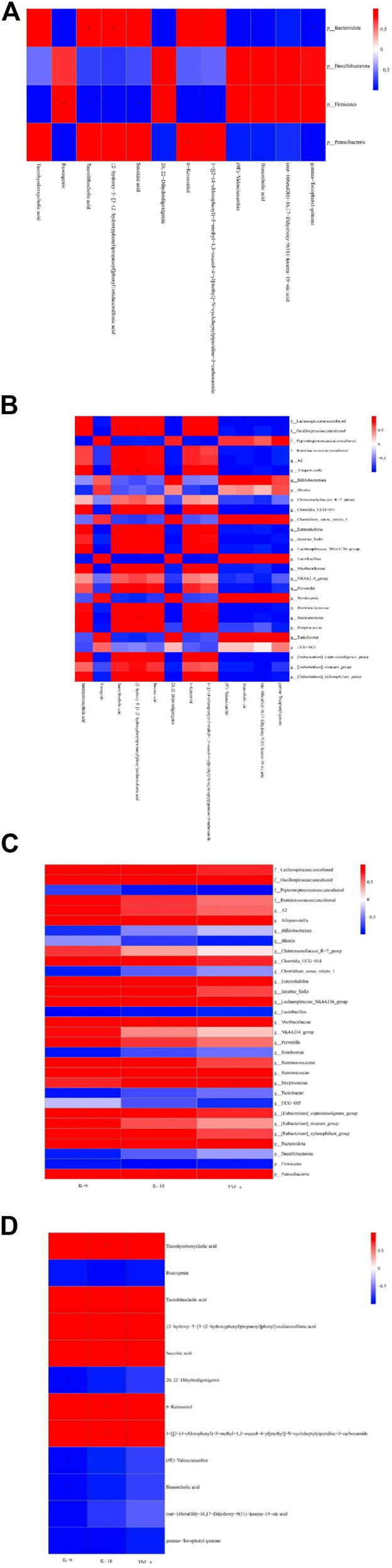
Pearson identified correlations between metabolites and the microbiome and inflammation indicators. **(A)** Phylum-level correlations between metabolites and microflora. **(B)** Genus-level correlations between metabolites and microflora. **(C)** Correlation between microflora and indicators of inflammation. **(D)** Correlation between metabolites and inflammation indicators.*p < 0.05 and **p < 0.01. Red indicates a positive correlation, blue indicates a negative correlation, bright color indicates a high correlation, and light color indicates a low correlation.

Pearson’s analysis found significant correlations between three variables, with criteria for significance including a correlation coefficient |r|≥0.7 and a *p*-value < 0.05. Patescibacteria at the phylum level was positively correlated with 6-ketoestriol and 1-[[2-(4-chlorophenyl)-5-methyl-1,3-oxazol-4-yl]methyl]-N-cycloheptylpiperidine-3-carboxamide. Firmicutes showed a positive correlation with ruscogenin and negative correlations with taurolithocholic acid and succinic acid (Figure 6A). Bacteroidota correlated positively with taurolithocholic acid and {2-hydroxy-5-[3-(2-hydroxyphenyl)propanoyl]phenyl}oxidanesulfonic acid, but negatively with gamma-tocopheryl quinone. At the genus level, gamma-tocopheryl quinone was negatively correlated with Enterorhabdus, uncultured_ Oscillospiraceae, and Alloprevotella. (ent-16betaOH)-16, 17-dihydroxy-9 (11)-kauren-19−oic acid was negatively correlated with Ruminococcaceae and Incertae_Sedis. Bisnorcholic acid was negatively correlated with [Eubacterium]_coprostanoligenes_group, Clostridia_UCG-014, and uncultured Lachnospiraceae (Figure 6B). (9e)-Valenciaxanthin exhibited a significant negative correlation with the abundance of [Eubacterium]_coprostanoligenes_group, Clostridia_UCG-014, and uncultured Lachnospiraceae. Similarly, 20,22-dihydrodigoxigenin showed a negative correlation with the same bacterial groups. Ruscogenin demonstrated an inverse relationship with Lachnospiraceae_NK4A136_group and Enterorhabdus, while 6-ketoestriol displayed a positive correlation with the abundance of Muribaculaceae. Succinic acid was positively correlated with both Muribaculaceae and Lachnospiraceae_NK4A136_group and Enterorhabdus. The concentration of {2-hydroxy-5-[3-(2-hydroxyphenyl) propanoyl] phenyl} oxidanesulfonic acid exhibited a positive association with the abundance of *Ruminococcus*, *Alloprevotella*, and uncultured Oscillospiraceae, while showing a negative correlation with *Lactobacillus*. Conversely, the level of taurolithocholic acid was positively linked with Lachnospiraceae_NK4A136_group, *Enterorhabdus*, *Alloprevotella*, and uncultured Oscillospiraceae.

We also observed that TNF-α had a negative correlation with uncultured Peptostreptococcaceae and a positive correlation with *Streptococcus*. IL-6 had a positive correlation with [Eubacterium] _ coprostanoligenes_group, Clostridia_UCG-014 PE, and uncultured Lachnospiraceae, and IL-1ß had a positive correlation with Muribaculaceae (Figures 6C,D). Further analysis found significant negative correlations between IL-6 and metabolites 20,22-dihydrodigoxigenin, (9E)-valenciaxanthin, and bisnorcholic acid. IL-1ß was positively correlated with taurohyodeoxycholic acid, 6-ketoestriol, and 1-[[2-(4-chlorophenyl)-5-methyl-1,3-oxazol-4-yl]methyl]−N−cycloheptylpiperidine-3-carboxamide, suggesting a link between changes in microbial community and its metabolites with inflammatory markers in the cecum (Cheng et al., 2022).

**FIGURE 7:**
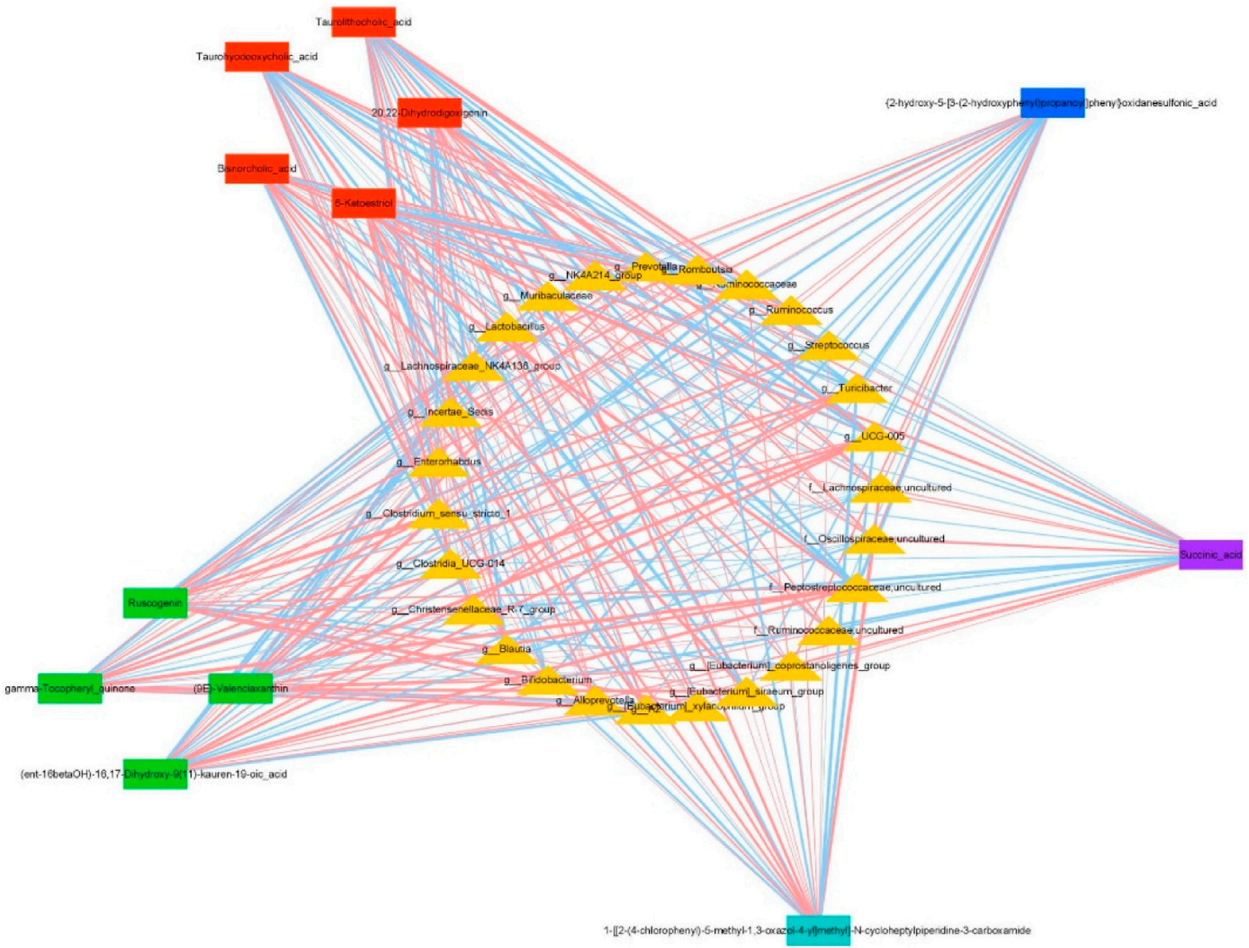
Co-expression networks show relationships between differentially expressed genera and metabolites under DYY treatment. Triangles represent genera, and rectangles represent metabolites. Red lines show positive correlations, and blue lines show negative correlations. Thicker lines indicate stronger correlations.

Based on above findings, we used Cytoscape 3.7.2 software to create a co-expression network involving 27 key intestinal flora and 12 key metabolites affected by DYY administration (Figure 7). Through analyzing the network, we speculated that *Turicibacter*, Oscillospiraceae_uncultured, UCG-005, Peptostreptococcaceae_uncultured, *Bifidobacterium*, and [Eubacterium]_coprostanoligenes_group as function-specific bacteria that may have a therapeutic impact in treating ALI with DYY. These findings indicate that these bacteria could potentially serve as markers to differentiate between healthy and ALI rats in a small sample size. DYY may have a beneficial impact on ALI treatment by reducing the abundance of *Turicibacter*, Oscillospiraceae_uncultured, and [Eubacterium]_coprostanoligenes_group bacteria and increasing the abundance of *Bifidobacterium*. However, further research with a larger sample size is necessary to confirm these results (Cai et al., 2024).

## 4 Discussion

Inflammatory responses in ALI, triggered by LPS, play a key role in causing damage to pulmonary cells and dysfunction (Li et al., 2022; Luo et al., 2021), detected by levels of inflammatory factors like TNF-α in BALF to reveal tissue lesions and lung function (Mokra and Kosutova, 2015; Tang et al., 2014; Guo et al., 2015). Injured endothelial cells release reactive oxygen species, causing inflammation and increased permeability in the lungs, leading to pulmonary edema. DYY and DXM were found to reduce inflammation and protect lung tissue in ALI rats, showing potential therapeutic benefits. Further research is needed to understand how DYY alleviates ALI (Cheng et al., 2023).

New research indicates that gut microbiota can affect health and disease susceptibility. Imbalance in gut microbiota can lead to diseases, including those affecting lung health (Zhou et al., 2021; Vandana et al., 2020). Regulating microbiota with dietary supplements or natural products may improve health outcomes (Yamashiro, 2017; Genda et al., 2018; Yi et al., 2022; Wang et al., 2023). Hence, the impact of DYY on lung health through gut microbiota and metabolites was studied to reveal the exact mechanism (Lv et al., 2021; Samuelson et al., 2015).

As shown through α and β diversity analysis, we found that DYY can improve ALI in rats by regulating cecal microflora composition and abundance (Lozupone et al., 2012; Wei et al., 2021). Although DYY did not fully restore intestinal flora richness reduced by LPS, it showed some improvement. Further analysis showed changes in microbiota linked to ALI progression with DYY intervention, such as increased Bacteroidetes and decreased Firmicutes in ALI rats, in line with Han et al.'s previous findings (Han et al., 2023). Clinical trials have shown that ALI patients have higher levels of Bacteroidetes and lower levels of Firmicutes compared to healthy individuals (Mukherjee and Hanidziar, 2018; Panzer et al., 2018). Firmicutes play a role in controlling systemic immunity (Jordan et al., 2023), and their reduced ratio to Bacteroidetes may be a signature feature of gut microbial imbalance during ALI (Groves et al., 2018; Di, 2021). After DYY treatment, the ratio of Firmicutes to *Bacteroides* was normalized, suggesting that these two bacteria in the gut may be key in DYY’s regulation of ALI (Qin et al., 2010).

We analyzed changes in gut microbiota associated with ALI at the genus level, focusing on the top 30 genera including Muribaculaceae, Prevotella, Alloprevotella, *Bacteroides*, and others within the phylum of thick-walled bacteria such as *Lactobacillus*, Roseburia, Ruminococcus, Blautia, *Streptococcus*, and Ruminococcaceae. Prevotella levels are linked to immune response, while a decrease in its presence indicates poor health (Dillon et al., 2014), *Bacteroides* is harmful in cases of septic ALI (Han et al., 2023), as supported by our research findings. The “gut–lung axis” facilitates communication through microbiome-dependent immune cells and inflammatory mediators (Gray et al., 2017), with inflammatory mediators originating from the gut potentially affecting the immune response in the lungs (He et al., 2017). Fortunately, DYY supplementation has been found to restore Firmicutes bacteria that exhibit resistance to pathogens (Zhao et al., 2023), thereby promoting a healthy immune balance and aiding in the defense against respiratory diseases. Moreover, probiotics such as *Lactobacillus* and Bifidobacterium can also provide protective effects for respiratory virus infection (Wypych et al., 2019). LEfSe analysis showed that DYY may modulate the Firmicutes/Bacteroidetes ratio to enhance granulocyte–macrophage colony-stimulating factor (GM-CSF) signal transduction and activate alveolar macrophages via extracellular signal-regulated kinase (ERK) specific signals, leading to increased ROS activity and reduced lung inflammation, ultimately contributing to the anti-ALI effect (Dillon et al., 2014). Thus, increasing gut probiotics and reducing harmful microbiome is important. This is mainly due to changes in purine metabolism, mismatch repair, D-glutamine and D-glutamate metabolism, and glycerolipid metabolism, as shown by PICRUSt analysis.

Research indicates that intestinal flora metabolites play a vital role in maintaining intestinal balance. After LPS induction, 12 metabolites showed significant changes, which were reversed by DYY treatment. This suggests that DYY may affect ALI through metabolite regulation, including pathways like purine metabolism, but we cannot ignore the regulatory effect of bile acid (BA) metabolism on ALI. BAs are a group of metabolites that are excreted by the host into the intestine and subsequently metabolized by intestinal microorganisms. Research indicates that BAs have the ability to modulate various physiological processes through interaction with the Takeda G protein-coupled receptor 5 (TGR5) membrane receptor (Chiang and Ferrell, 2020), which is highly expressed in macrophages. Upon binding to its ligand, TGR5 activates multiple kinase pathways including PKA, AKT, SRC kinase, and ERK1/2. This activation plays a role in regulating glucose homeostasis, energy metabolism, macrophage-mediated inflammation, and maintaining intestinal immune homeostasis (Kida et al., 2013; Maruyama et al., 2002; Hu et al., 2019; Eblimit et al., 2018). Taurolithocholic acid (TLCA), a secondary bile acid originating from chenodeoxycholic acid (CDCA), is recognized as a potent natural agonist of TGR5 and possesses anti-inflammatory properties (Dosa et al., 2013; Ko et al., 2019). In a similar vein, taurohyodeoxycholic acid (THDCA), a bile acid with a 6α-hydroxyl group, has been shown to exhibit anti-inflammatory effects in the intestines by reducing the secretion of Th1/Th17-related cytokines such as interferon-γ, IL-6, IL-17A, IL-21, IL-22, and TNF-α (Lv et al., 2023). In this research, the cecal abundance of taurohyodeoxycholic acid and taurolithocholic acid in LPS-induced rats was found to be higher compared to the control group, while the abundance of bisnorcholic acid was lower. These findings indicate that the inflammatory response triggered by LPS may stimulate the change of the aforementioned metabolites to exert anti-inflammatory effects. Following treatment with DYY, the concentrations of these metabolites returned to normal levels.

Moreover, recent research suggests that succinic acid, a byproduct of gut microbiota metabolism, may contribute to the development of lung disorders by affecting the immune response. Studies have shown that succinic acid can trigger programmed cell death in alveolar epithelial cells by promoting the polarization of alveolar macrophages toward a pro-inflammatory M1 phenotype. This is believed to occur through SUCNR1 receptor and PI3K/AKT/HIF-1 α signaling pathway, shedding light on the mechanism of gut-derived ALI through the lens of the “gut–lung axis” (Wang and Li, 2023). Evidence indicates that ruscogenin (RUS) protects against LPS-induced pulmonary endothelial barrier disruption by targeting mediating non-muscle myosin heavy chain IIA (NMMHC IIA)‒Toll-like receptor 4 (TLR4), reducing apoptosis of pulmonary endothelial cells, and attenuating pulmonary edema (Wu et al., 2022; Wu et al., 2020). Rats in the LPS group had higher succinic acid and lower RUS levels in the cecum, but DYY reduced lung injury by decreasing succinate and increasing RUS production.

Correlation analysis found that DYY improves ALI by affecting gut bacteria levels (Liu et al., 2023), with Firmicutes positively correlated with ruscogenin and negatively correlated with succinic acid. Bacteroidota showed opposite correlations, suggesting DYY promotes beneficial bacteria and reduces harmful bacteria. This highlights the two-way connection between gut bacteria and lung health in the intestinal–lung axis (Samuelson et al., 2015). This axis facilitates the passage of various substances, such as endotoxins, cytokines, intestinal bacteria, and their metabolites like short-chain fatty acids (SCFA), from the intestines to the lungs and ultimately into the bloodstream (Dumas et al., 2018). Prevotella, a significant bacterium in this process, is known for its ability to synthesize SCFA via the acetyl-CoA pathway utilizing pyruvate as a precursor (Louis et al., 2014; Neves et al., 2020). The SCFA produced is metabolized in the gastrointestinal tract and then transported to the liver for further processing, with a portion eventually reaching the lungs through the intestinal–lung axis. This pathway has been shown to impact immune cell differentiation and maturation in lung tissue and regulating pulmonary inflammation (Anand and Mande, 2018). SCFAs are also produced by various bacteria like Bifidobacterium, *Streptococcus*, *Lactobacillus*, and Lachnospiraceae_NK4A136_group (Layden et al., 2013; Yan et al., 2023) and play a crucial role in intestinal health and disease prevention, serving as a key link between diseases, nutrients, and gut flora (Nicolas and Chang, 2019). This study identified a negative correlation between Prevotella and *Streptococcus* with ruscogenin and a positive correlation with succinic acid, as well as a contrasting relationship between *Lactobacillus* and Bifidobacterium. These findings suggest a potential impact by DYY treating on SCFA production and mitigation of inflammatory damage. Additionally, Firmicutes exhibited a negative correlation with inflammatory markers, while Bacteroidetes showed a positive correlation. *Lactobacillus* and Bifidobacterium were negatively correlated with inflammatory markers. Bifidobacterium may play a protective role in lung tissue by regulating SCFA production and inflammation through the “gut microbiota–gut–lung axis.” Succinic acid promotes inflammation, while ruscogenin inhibits it, as shown by correlations with inflammatory markers. Taurolithocholic acid (TLCA) has been shown to decrease inflammation through the inhibition of the NF-κB pathway and the secretion of cytokines such as TNF-α and IL-6 (Wang et al., 2021), aligning with the findings of this study. In short, DYY effectively ameliorates ALI by modulating intestinal flora and metabolites, similar to the previous research, but metabolic pathways differ slightly (Chen et al., 2022). These results suggest potential complex interactions between TCM and intestinal flora, warranting further investigation (Chen et al., 2019).

However, the study is limited in identifying the exact microbiome or metabolite responsible for ALI improvement with DYY. Further research is needed to understand the role of pulmonary microbiota and metabolites in ALI and how pulmonary microbes affect lung injury in DYY treatment (Lu et al., 2023).

## 5 Conclusion

Our research suggests a possible link between alterations in intestinal flora and metabolites and the development of ALI, highlighting the potential of DYY in treating this condition by modulating the “gut–lung axis.” Further study on lung microbial sequencing and intestinal barrier function is necessary to fully understand DYY’s impact on ALI treatment (Lu et al., 2023).

## Data Availability

The data presented in the study are deposited in the Metabolights repository, accession number MTBLS10741.
