# Supplementary figures and images for: Combined analysis of cecal microbiota and metabolomics reveals the intervention mechanism of Dayuan Yin in acute lung injury
Source: Front Pharmacol. 2024 Sep 10;15:1436017. doi: 10.3389/fphar.2024.1436017 (PMC11420052; doi:10.3389/fphar.2024.1436017)

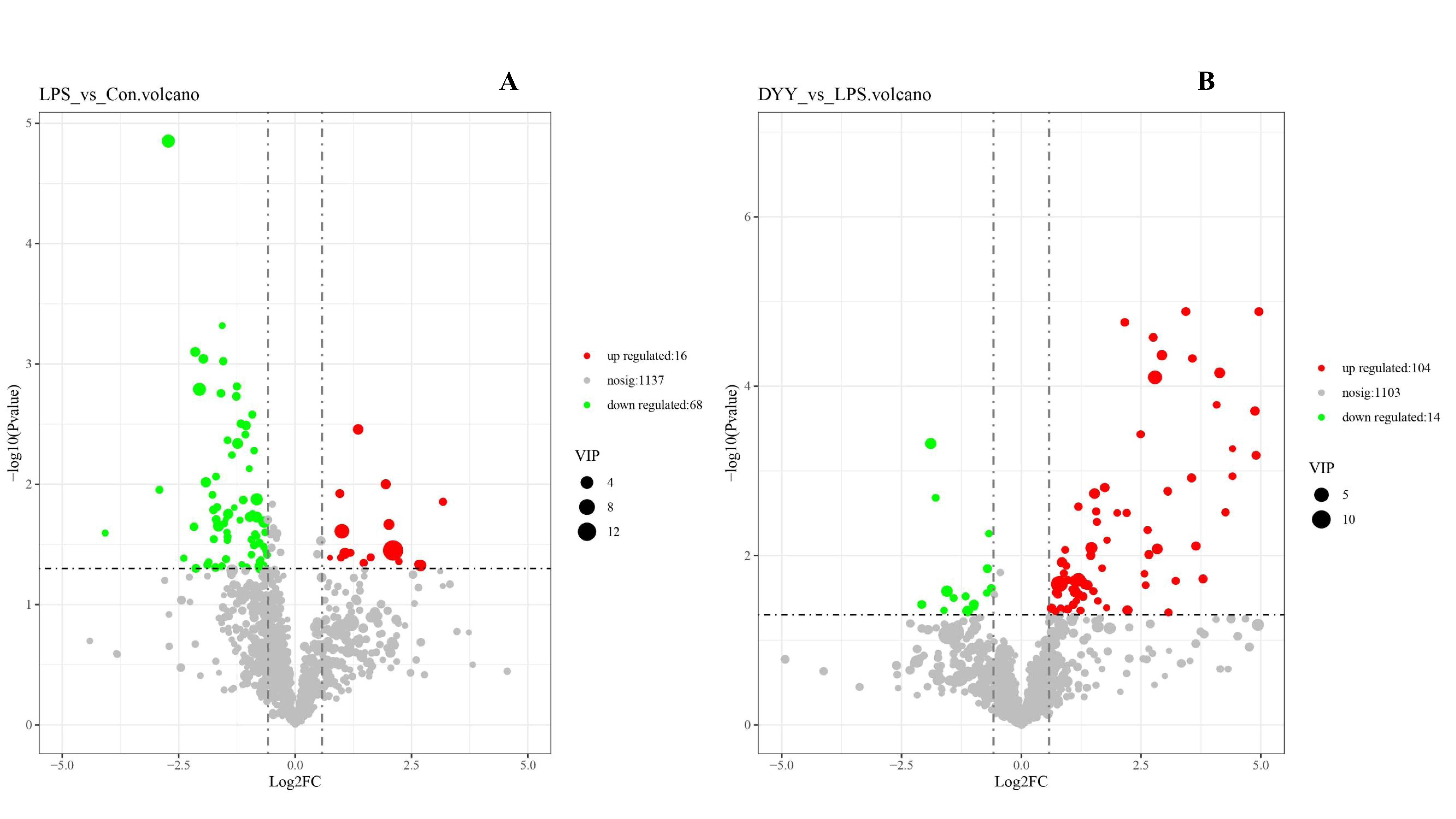

Supplement: Supplementary file 1 [file Image3.JPEG]

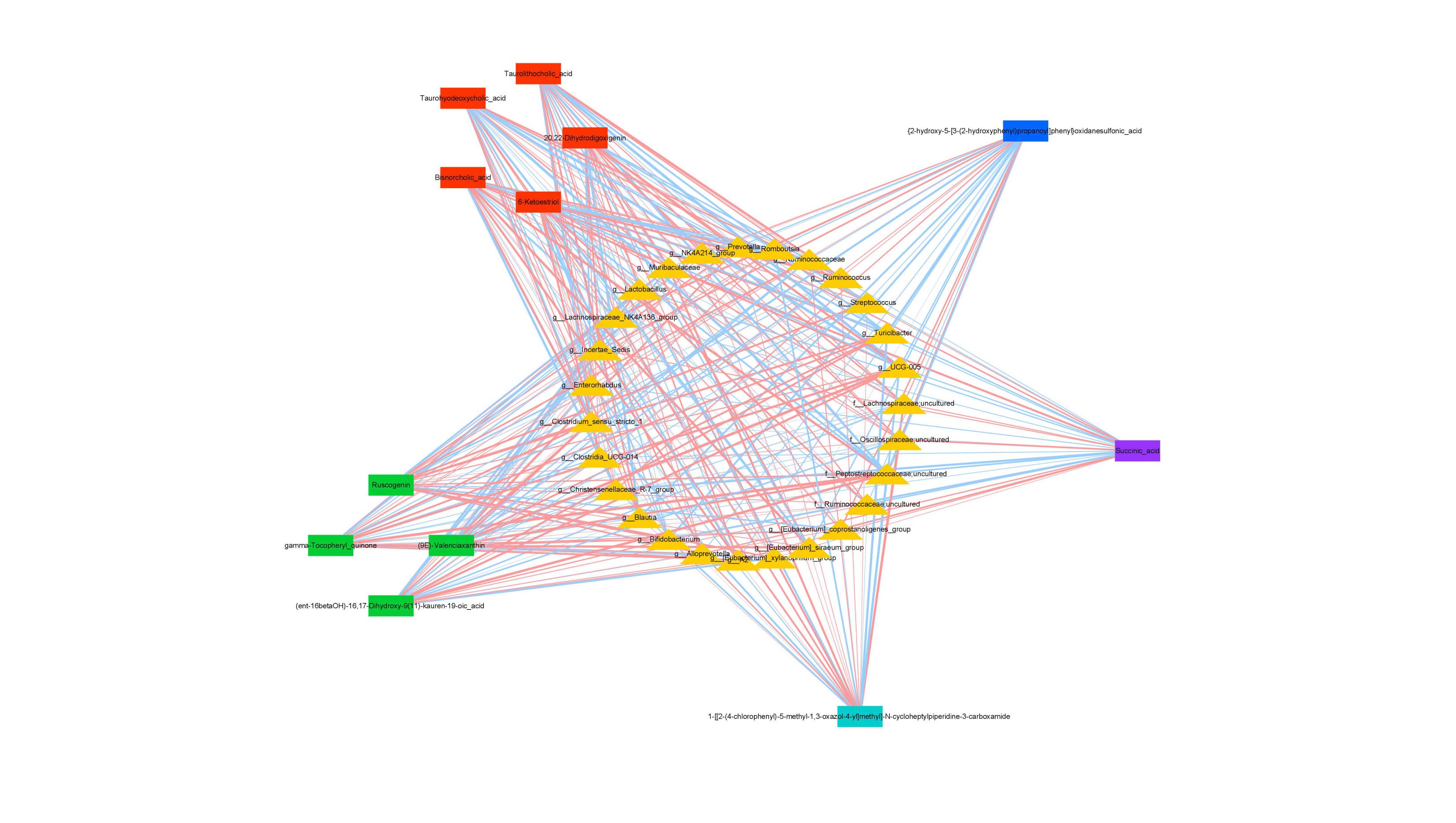

Supplement: Supplementary file 2 [file Image9.JPEG]

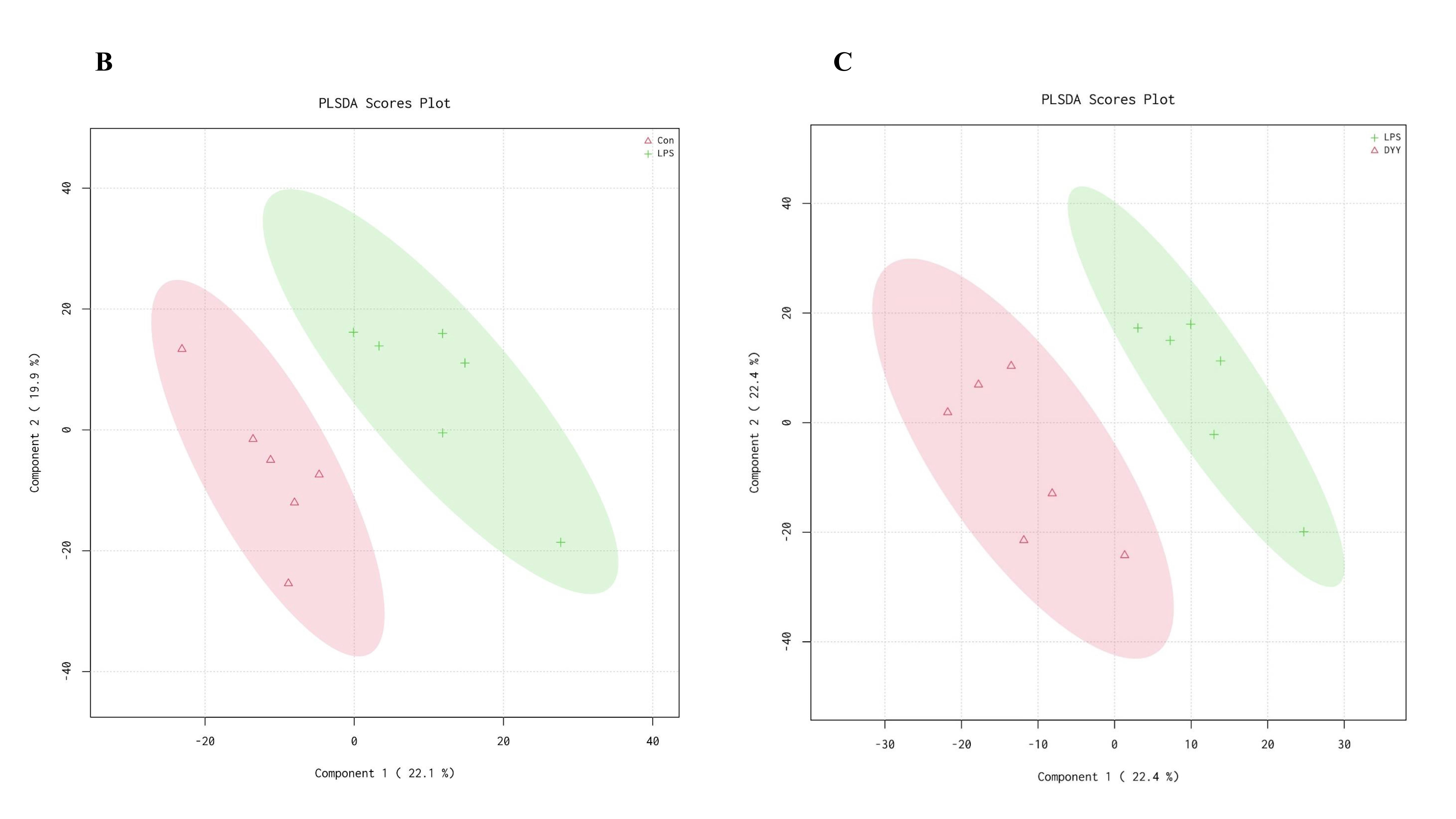

Supplement: Supplementary file 3 [file Image1.JPEG]

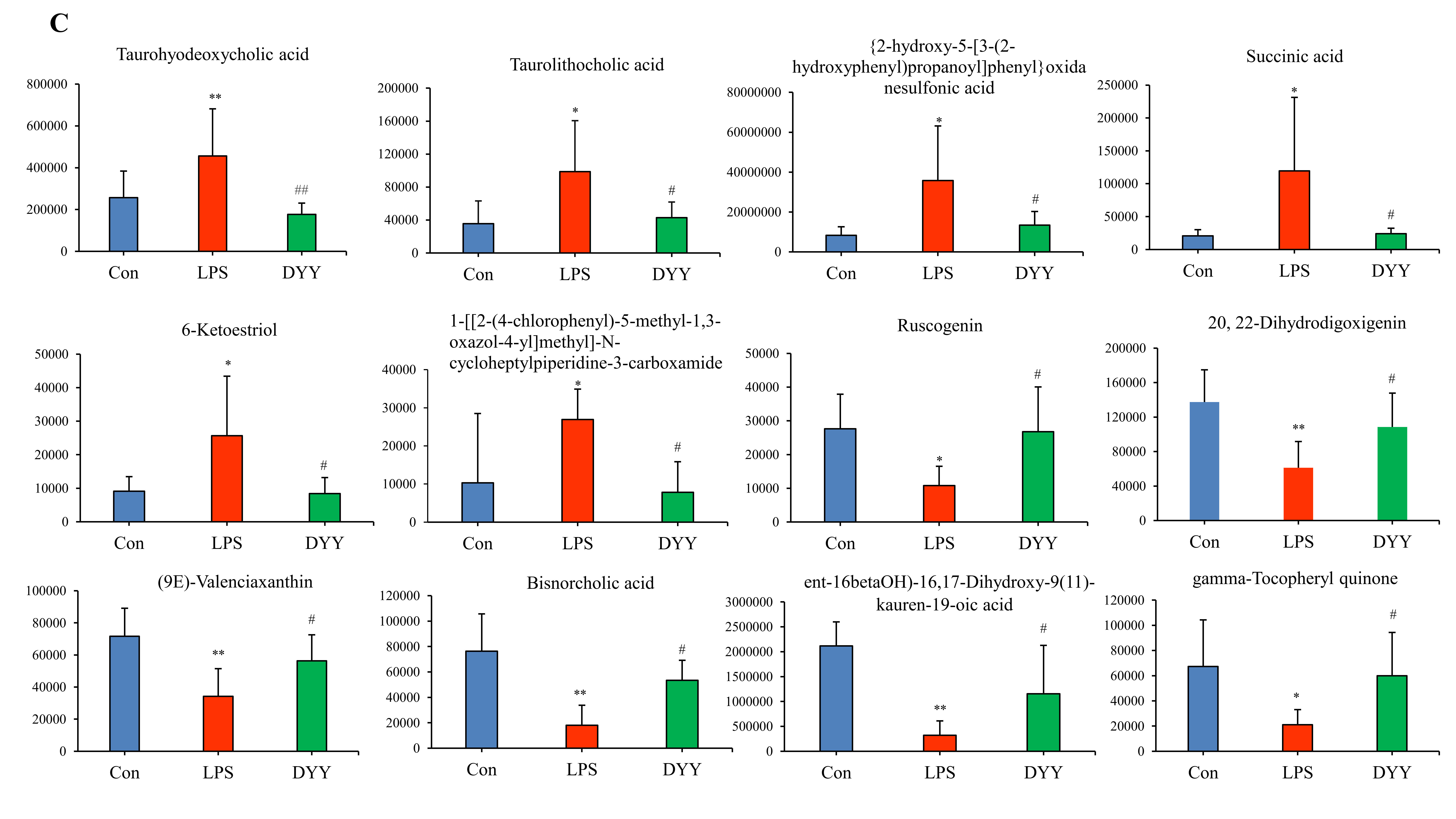

Supplement: Supplementary file 4 [file Image4.JPEG]

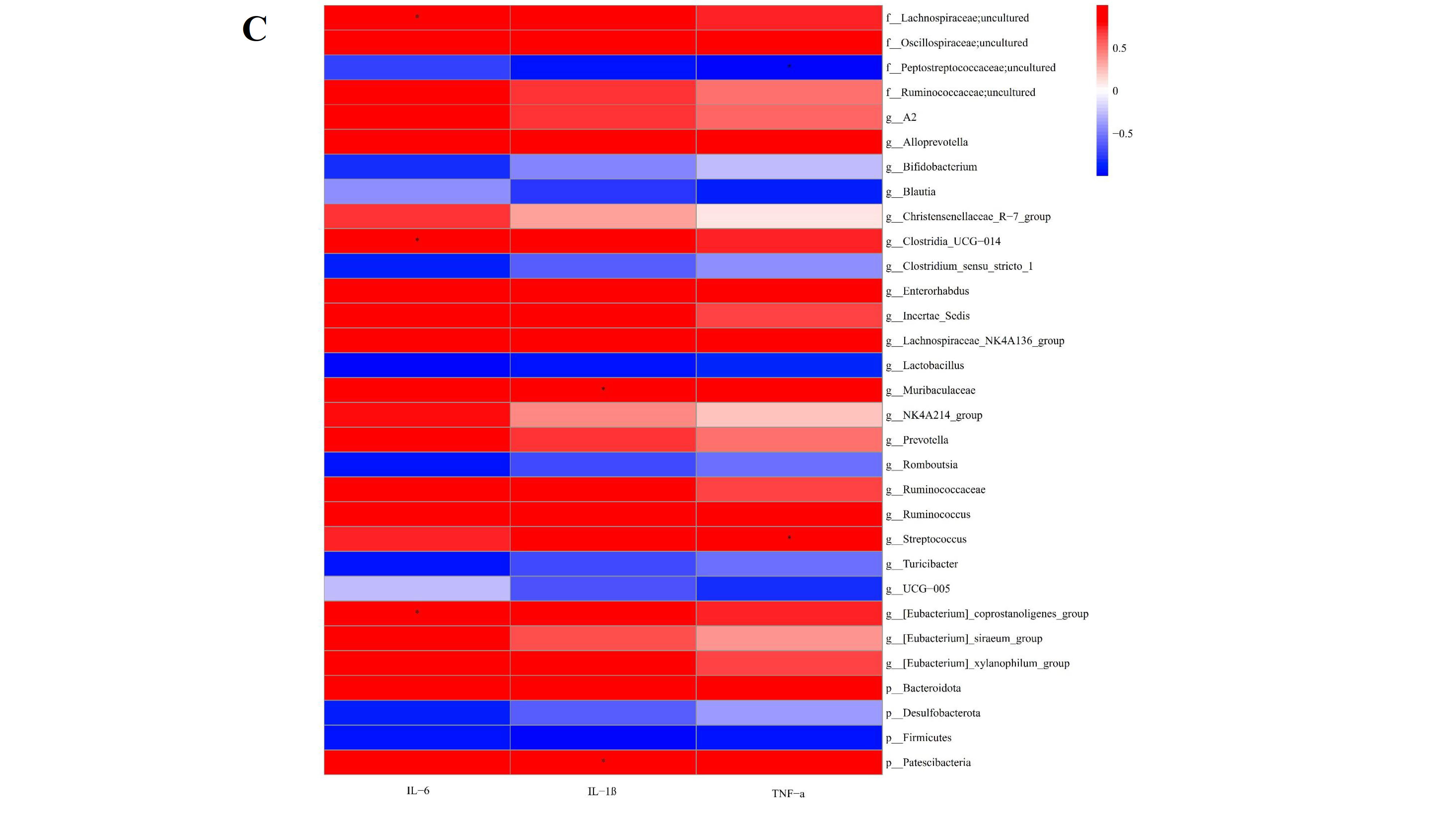

Supplement: Supplementary file 5 [file Image7.JPEG]

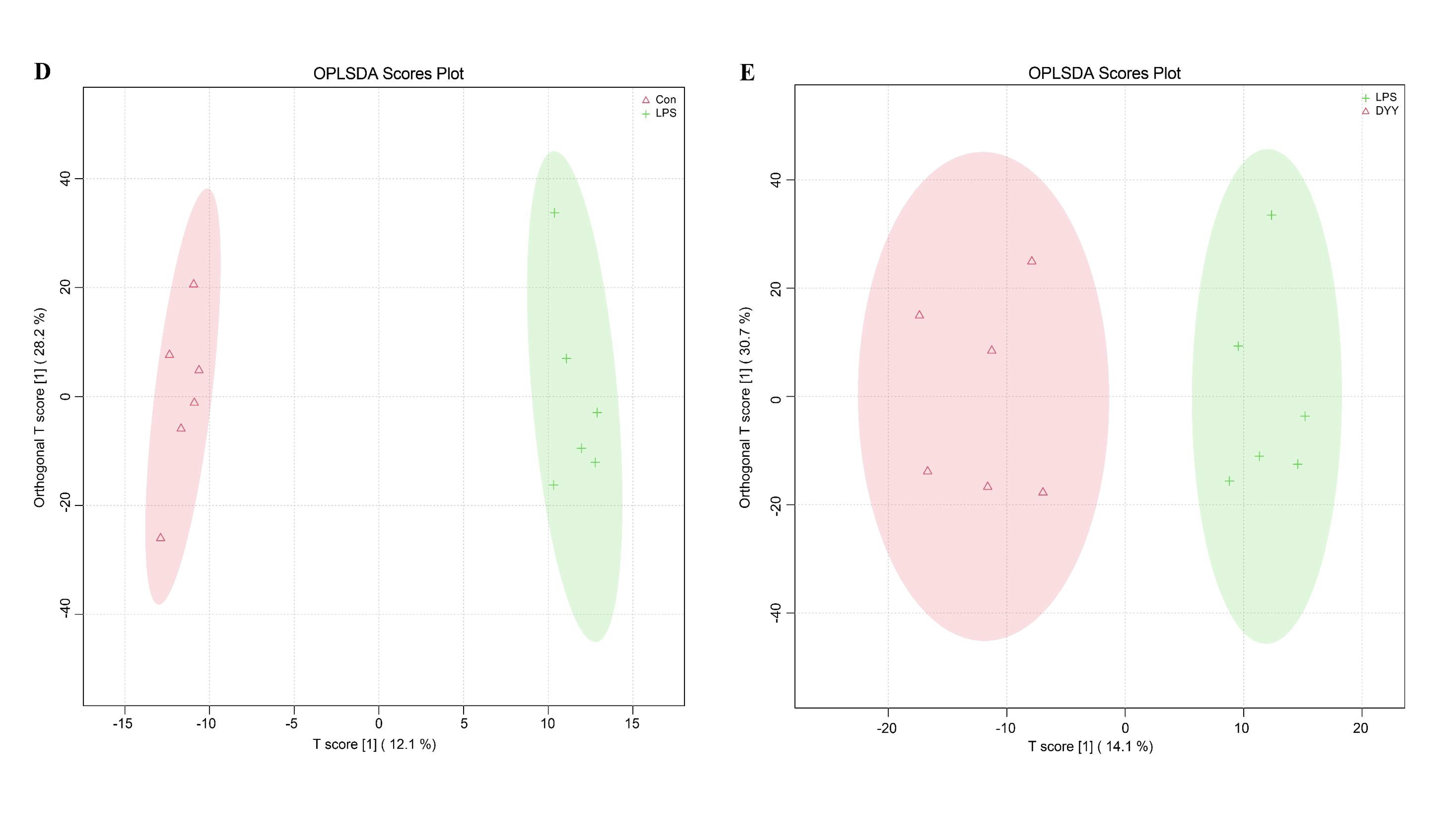

Supplement: Supplementary file 6 [file Image2.JPEG]

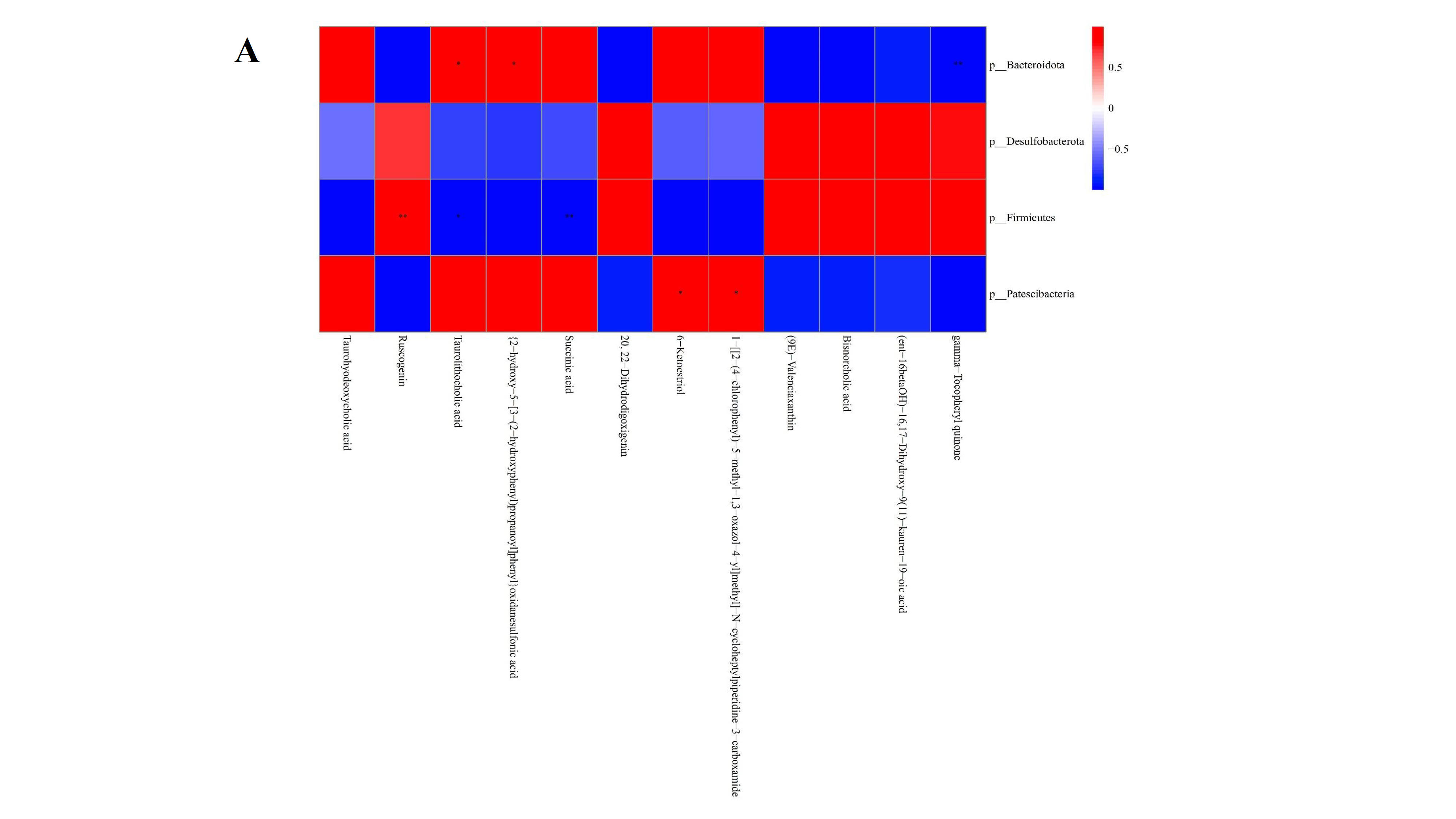

Supplement: Supplementary file 7 [file Image5.JPEG]

ConVS LPS


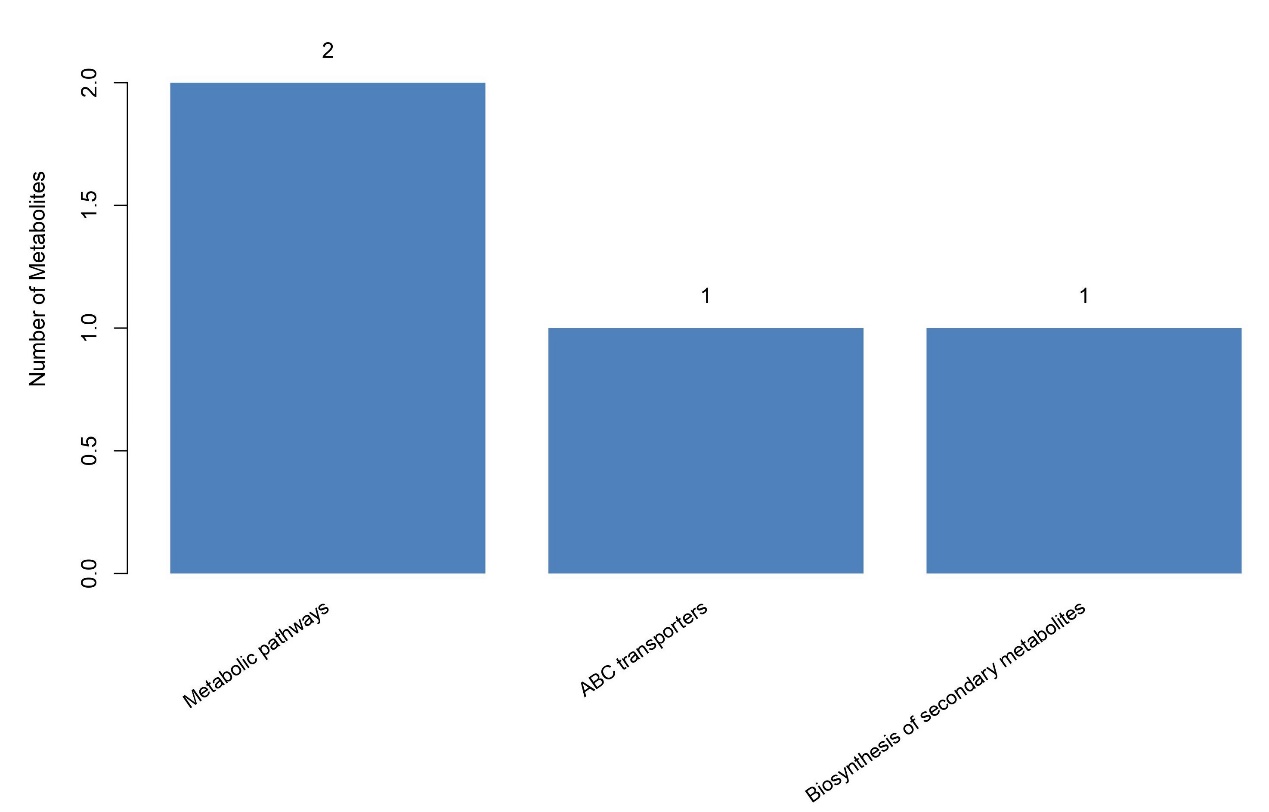


LPS VS DYY


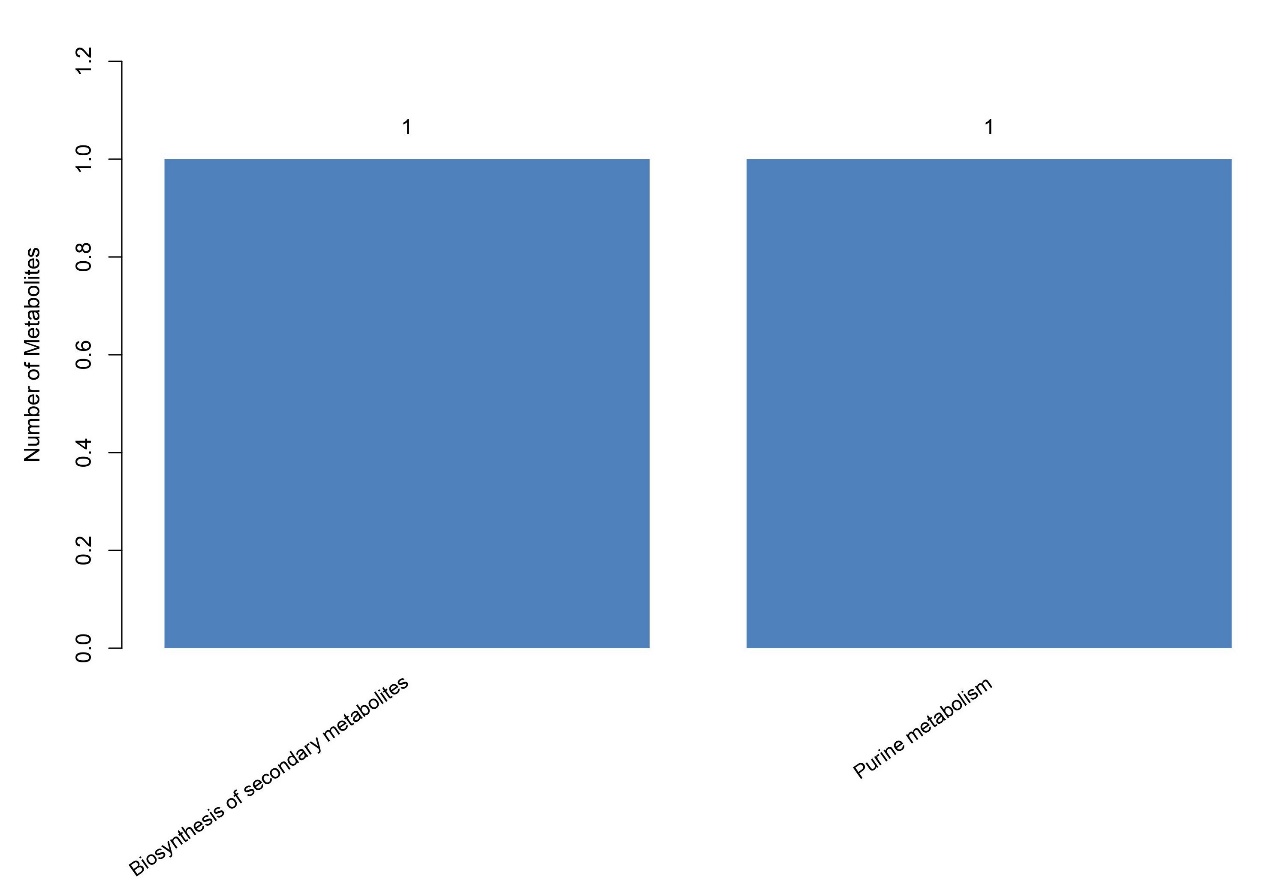


kegg_enrichment_bubble


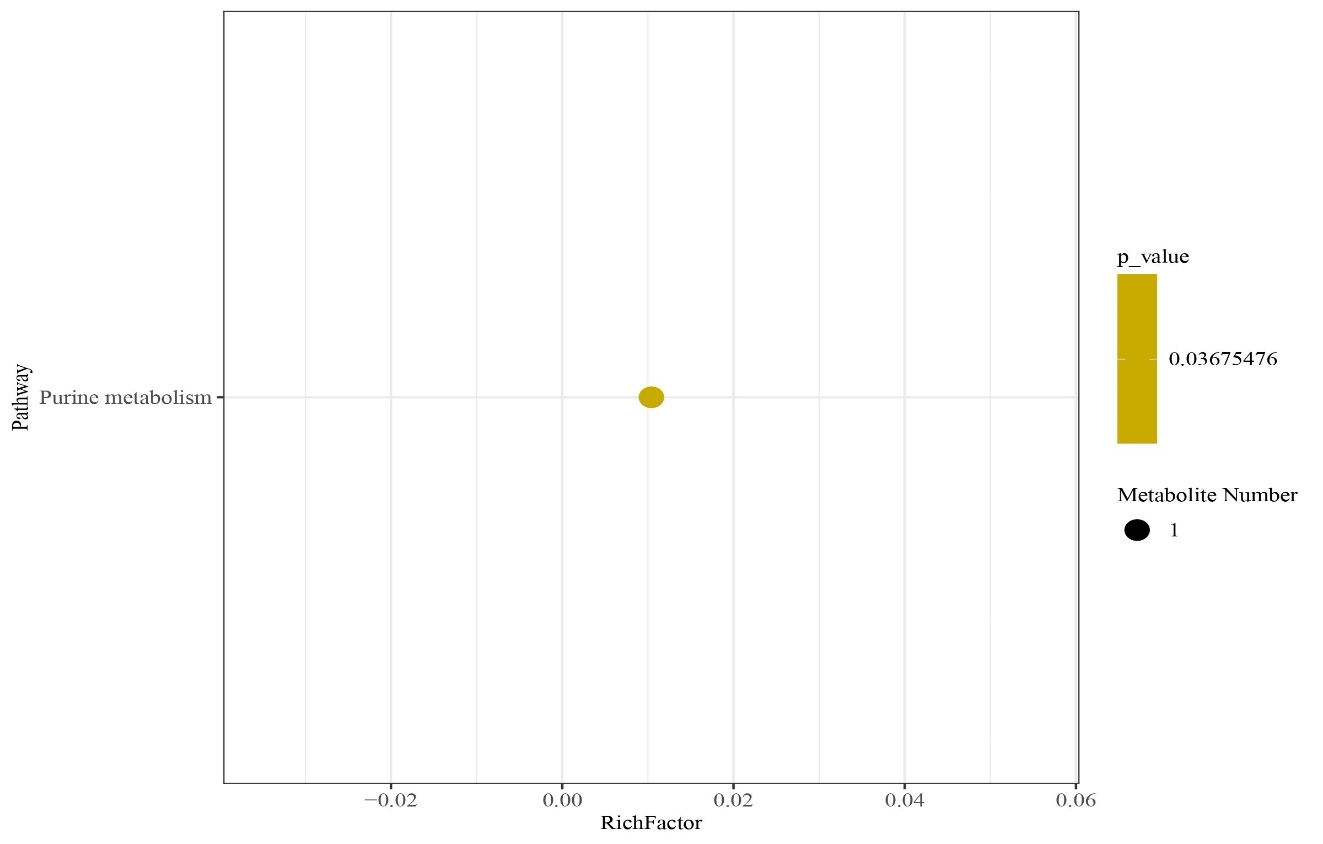


kegg_topology


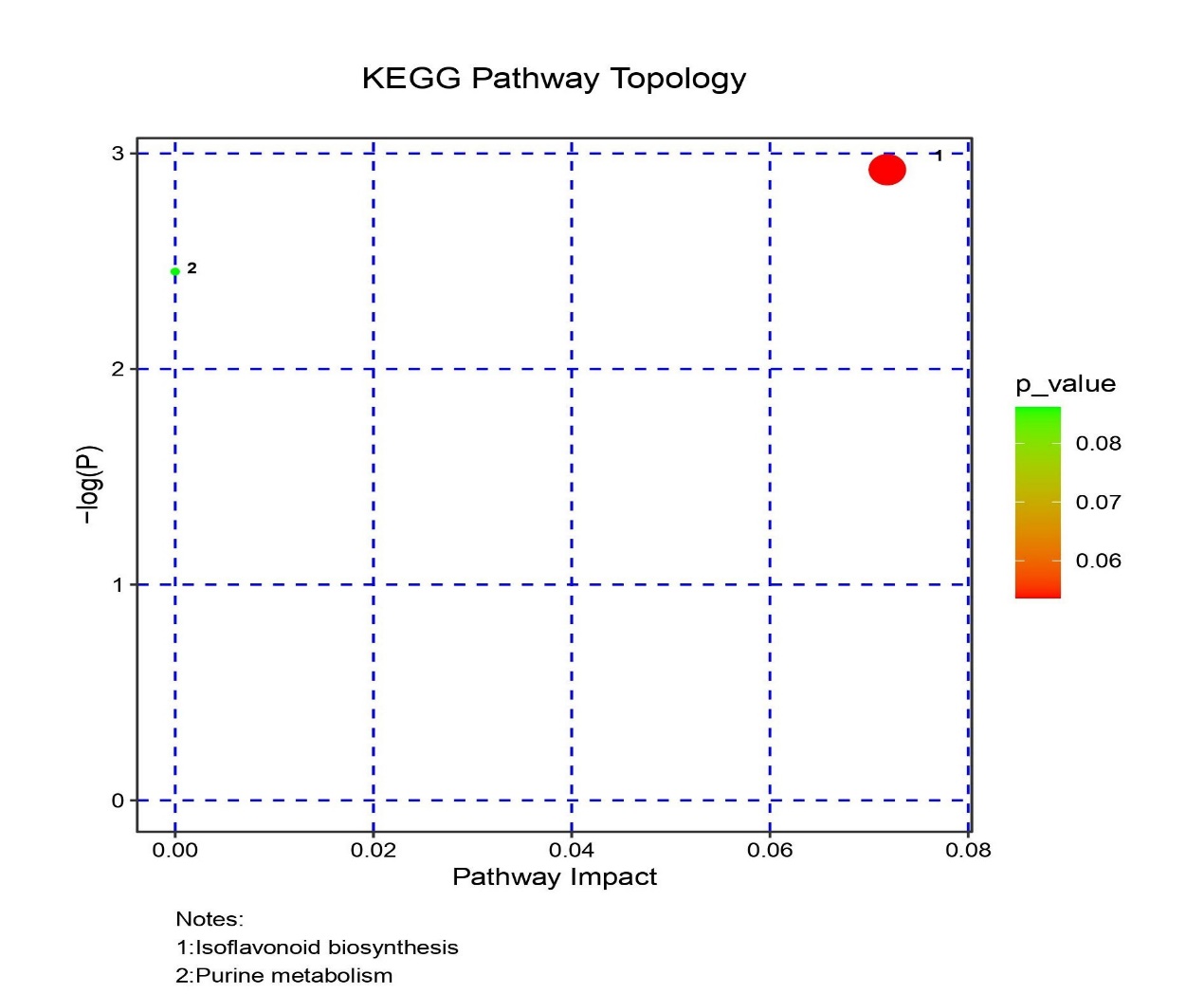

Supplement: Supplementary file 9 [file DataSheet2.docx]

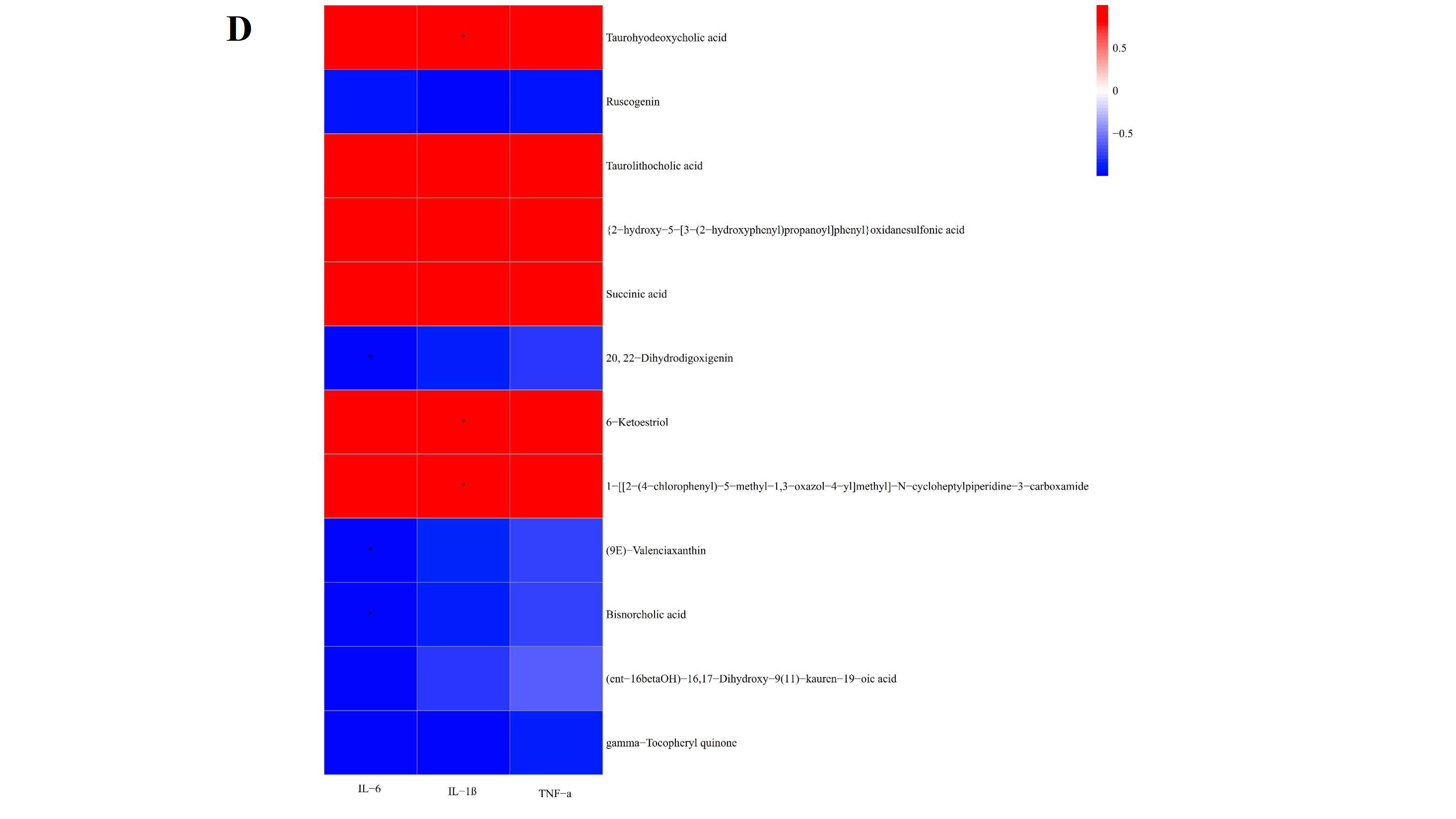

Supplement: Supplementary file 10 [file Image8.JPEG]

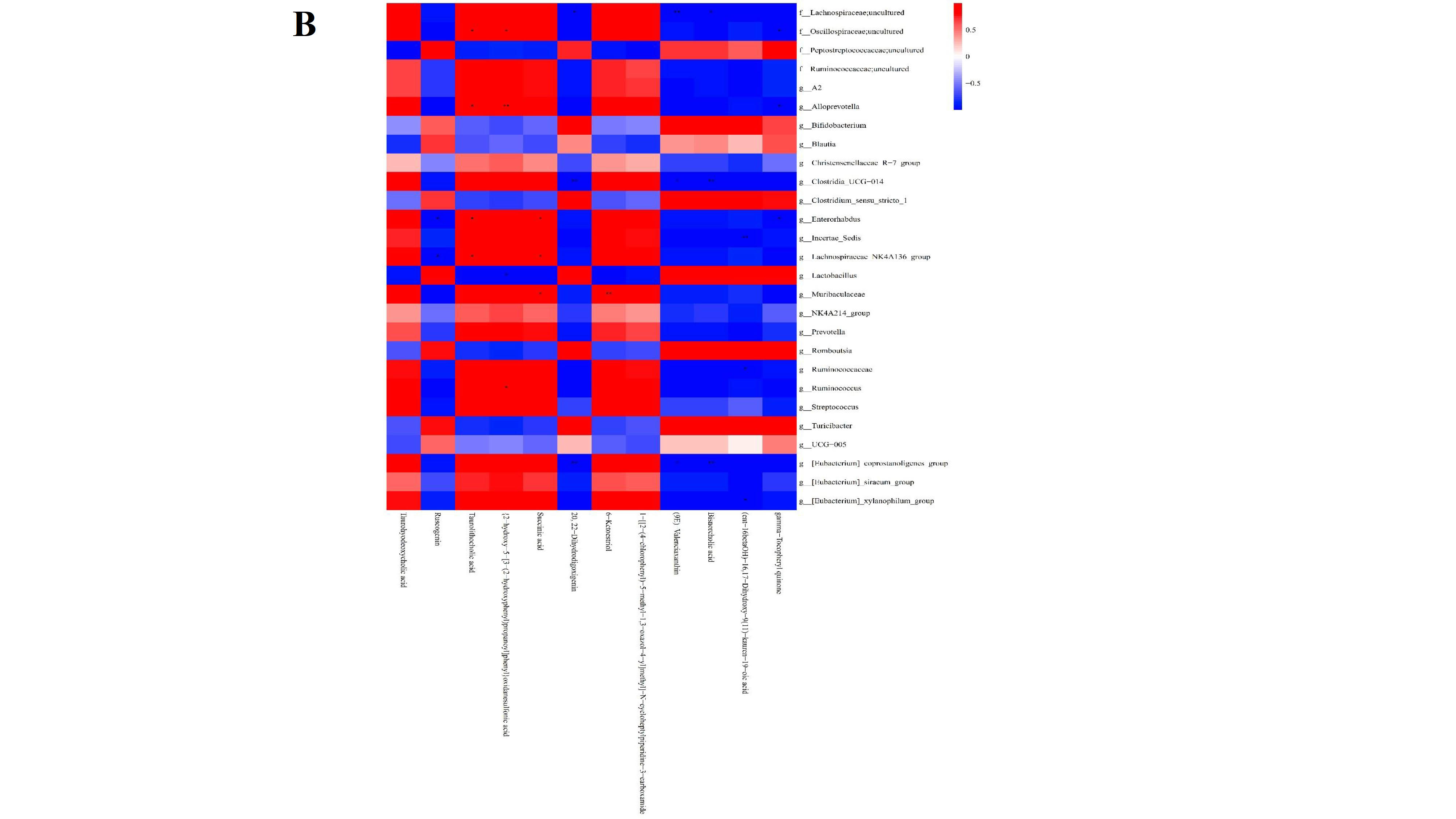

Supplement: Supplementary file 12 [file Image6.JPEG]
