# Supplementary material for: Combined analysis of cecal microbiota and metabolomics reveals the intervention mechanism of Dayuan Yin in acute lung injury
Source: Front Pharmacol. 2024 Sep 10;15:1436017. doi: 10.3389/fphar.2024.1436017 (PMC11420052; doi:10.3389/fphar.2024.1436017)
Supplement: Supplementary file 11 [file DataSheet1.docx]

**Abstract:** **Ion flow profiles in two scan modes in the LC-MS untargeted metabolome study.**

Title 1 Negative ion pattern of QC sample 1 in non-targeted metabolomics.

Title 2 Positive ion mode of QC sample 1 in non-targeted metabolomics.

Title 3 Negative ion pattern of QC sample 2 in non-targeted metabolomics.

Title 4 Positive ion mode of QC sample 2 in non-targeted metabolomics.

Title 5 Negative ion pattern of QC sample 3 in non-targeted metabolomics.

Title 6 Positive ion mode of QC sample 3 in non-targeted metabolomics.
